# Sports Medicine - Open

**DOI:** 10.1186/s40798-014-0001-9

**Published:** 2015-01-20

**Authors:** Roger Olney, Steve McMillan

**Affiliations:** Auckland, New Zealand

**Dear Sports Medicine and Exercise Researchers,**

It is with great pleasure that Springer announces the launch of *Sports Medicine* - *Open*, a new peer-reviewed, online-only, open-access journal designed to provide an avenue for publication of original research in the field of sports and exercise medicine.

*Sports Medicine* - *Open* is a sister publication of *Sports Medicine*, which has been published since 1984 by Adis and is now part of the Springer journal portfolio. The original *Sports Medicine* journal has been a leading publication in sports and exercise medicine for 30 years and has consistently been one of the most highly ranked vehicles for publication of review articles in the field. This focus on reviews has left little scope for the inclusion of original research, although *Sports Medicine* regularly receives submissions of original research articles. The launch of a sister journal to *Sports Medicine,* publishing original research, aims to provide the first top-tier open-access avenue for these papers. Given the well-regarded *Sports Medicine* brand and the community of top-tier researchers that support the journal, we believe that *Sports Medicine* - *Open* will fill a gap for our target audience of sports medicine practitioners, physiotherapists, exercise scientists/physiologists, team doctors, and trainers.

*Sports Medicine* - *Open* will have similar aims and scope to those of the original *Sports Medicine* journal but with a different, complementary focus. Specifically, *Sports Medicine* - *Open* aims to publish original medical and scientific research in the following areas:

- Sporting performance enhancement
- Medical syndromes associated with sport and exercise
- Injury prevention and treatment
- Exercise for rehabilitation and health
- The application of physiological and biomechanical principles to specific sports

*Sports Medicine* - *Open* will also consider publication of a smaller number of high-interest, definitive reviews. To ensure consistency and continuity of the editorial services associated with the original *Sports Medicine* journal, *Sports Medicine* - *Open* will be managed by an expanded team based on the editorial staff responsible for the original journal. Thus, authors will enjoy the benefits of publishing in a journal following the strong publishing practices of *Sports Medicine,* with the same editorial and publishing expertise and experience associated with that journal.

Springer publishes more than 2,200 journals, including over 500 in its Medicine Journal Collection. The successful integration of the Adis portfolio of journals into the Springer production systems and custom-built platforms (SpringerLink, Springer for R&D, and Springer for Hospitals & Health) means that authors now have:

- Easy article submission, peer review, and tracking processes
- Shorter time to publication
- Unparalleled article visibility, worldwide reach and discoverability
- Convenient, global, online access of readers to their work

These benefits of the Springer organization will flow into the new *Sports Medicine* - *Open* journal from its inception.

Therefore, our final message is a simple one: if you have an original research article in the sports and exercise field that you believe would be a candidate for publication in *Sports Medicine* - *Open*, please submit your manuscript to us for an assessment. With the support of both the community of researchers who publish review articles in the original *Sports Medicine* journal, and the large number of other readers and followers of the journal, we believe that we can quickly establish a journal that provides a high-quality, open-access option for researchers in the field. We ask you, particularly if you are a ‘friend’ of the original *Sports Medicine* journal, to keep *Sports Medicine* - *Open* in mind when considering where to submit your original research.

Yours sincerely

Roger Olney and Steve McMillan, Editors

editorial@sportsmedicine-open.com

